# A Proteomic Platform Enables to Test for AML Normalization *In Vitro*

**DOI:** 10.3389/fchem.2022.826346

**Published:** 2022-02-01

**Authors:** Samuel M. Meier-Menches, Benjamin Neuditschko, Lukas Janker, Marlene C. Gerner, Klaus G. Schmetterer, Albrecht Reichle, Christopher Gerner

**Affiliations:** ^1^ Department of Analytical Chemistry, Faculty of Chemistry, University of Vienna, Vienna, Austria; ^2^ Institute of Inorganic Chemistry, Faculty of Chemistry, University of Vienna, Vienna, Austria; ^3^ Joint Metabolome Facility, University of Vienna and Medical University of Vienna, Vienna, Austria; ^4^ Department of Laboratory Medicine, Medical University of Vienna, Vienna, Austria; ^5^ Division of Biomedical Science, University of Applied Sciences FH Campus Wien, Vienna, Austria; ^6^ Department of Internal Medicine III, Haematology and Oncology, University Hospital Regensburg, Regensburg, Germany

**Keywords:** AML—acute myeloid leukaemia, arsenic trioxide, cancer, differentiation, normalization, plecstatin-1, proteomics, Ruthenium

## Abstract

Acute promyelocytic leukaemia (APL) can be cured by the co-administration of arsenic trioxide (ATO) and all-*trans* retinoic acid (ATRA). These small molecules relieve the differentiation blockade of the transformed promyelocytes and trigger their maturation into functional neutrophils, which are physiologically primed for apoptosis. This normalization therapy represents a compelling alternative to cytotoxic anticancer chemotherapy, but lacks an *in vitro* model system for testing the efficiency of novel combination treatments consisting of inducers of differentiation and metallopharmaceuticals. Here, using proteome profiling we present an experimental framework that enables characterising the differentiation– and metal-specific effects of the combination treatment in a panel of acute myeloid leukaemia (AML) cell lines (HL-60 and U937), including APL (NB4). Differentiation had a substantial impact on the proteome on the order of 10% of the identified proteins and featured classical markers and transcription factors of myeloid differentiation. Additionally, ATO provoked specific cytoprotective effects in the AML cell lines HL-60 and U937. In HL-60, these effects included an integrated stress response (ISR) in conjunction with redox defence, while proteasomal responses and a metabolic rewiring were observed in U937 cells. In contrast, the APL cell line NB4 did not display such adaptions indicating a lack of plasticity to cope with the metal-induced stress, which may explain the clinical success of this combination treatment. Based on the induction of these cytoprotective effects, we proposed a novel metal-based compound to be used for the combination treatment instead of ATO. The organoruthenium drug candidate plecstatin-1 was previously shown to induce reactive oxygen species and an ISR. Indeed, the plecstatin-1 combination was found to affect similar pathways compared to the ATO combination in HL-60 cells and did not lead to cytoprotective response signatures in NB4. Moreover, the monocytic cell line U937 showed a low plasticity to cope with the plecstatin-1 combination, which suggests that this combination might achieve therapeutic benefit beyond APL. We propose that the cytoprotective plasticity of cancer cells might serve as a general proxy to discover novel combination treatments *in vitro*.

## Introduction

Metals in medicine form an important pillar of cytotoxic chemotherapy in cancer treatments since the discovery of cisplatin (Rosenberg et al., 1969; Kelland, 2007). Driven by its success, this strategy was transferred to next-generation platinum complexes and also to non-platinum anticancer agents (Casini et al., 2019). Prominent examples of the latter include the ruthenium(III) coordination complexes BOLD-100 (NKP-1339, IT-139) and TLD1433 or the gold(I) complex auranofin, which are currently under clinical evaluation (Bertrand and Casini, 2014; Trondl et al., 2014; Meier-Menches et al., 2018; Casini et al., 2019). Similarly, many promising investigational drug candidates are designed as cytotoxic agents using a variety of different metals (Casini et al., 2019; Franz and Metzler-Nolte, 2019).

**FIGURE 1:**
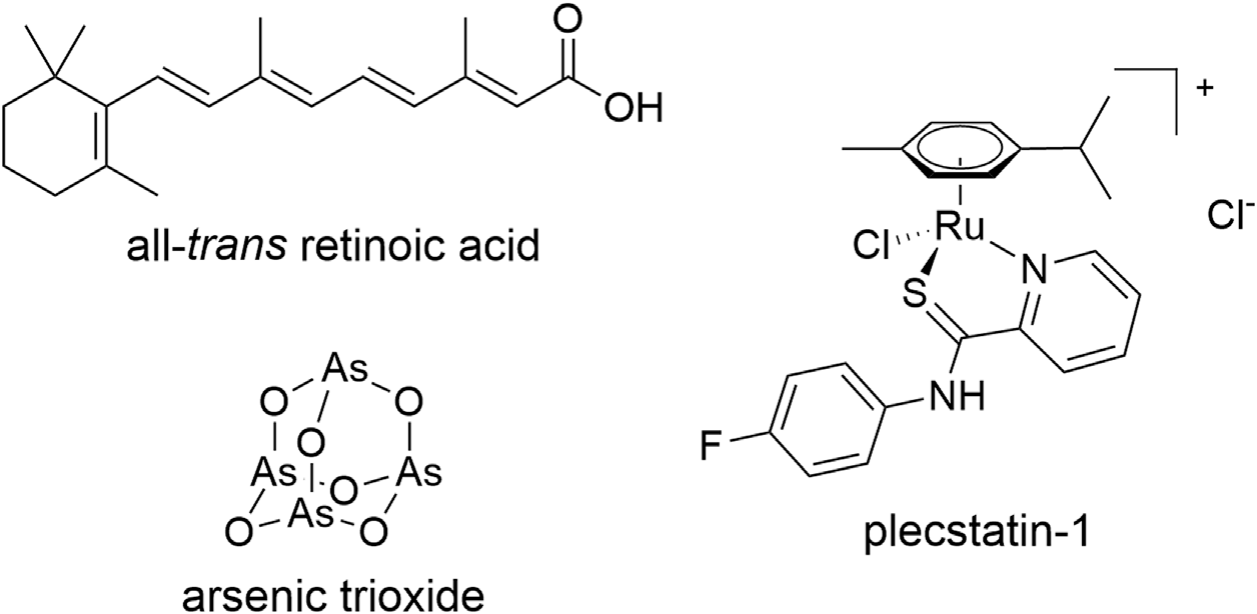
Chemical structures of the compounds used in this study.

Arsenic trioxide (ATO, Figure 1) is an intriguing example of the small number of clinically approved anticancer metal(-loid)s. It is used in combination with all-*trans* retinoic acid (ATRA, Figure 1) to treat patients suffering from acute promyelocytic leukaemia (APL) and achieves cure rates of >90% (Lo-Coco et al., 2013; Burnett et al., 2015). The combined administration of ATO + ATRA shows synergistic effects by dual-targeting of promyelocytic leukaemia protein (PML)–retinoic acid receptor alpha (RARα), the expression product of the characteristic t(15,17) translocation, which is responsible for the differentiation blockade in APL (Testa and Lo-Coco, 2015; de Thé, 2018). In contrast to cytotoxic anticancer chemotherapy in which the therapeutic agents are administered at maximum tolerable doses, ATO + ATRA is administered in a low-dose treatment regime over a prolonged time period (Lo-Coco et al., 2013; Burnett et al., 2015; Heudobler et al., 2019b). The combination treatment leads to differentiation of cancerous promyelocytes into short-lived neutrophils and restores their physiological apoptosis program (Tak et al., 2013). Thus, the endpoint of this therapy is not apoptosis induction, but a remodelling of the cancer cells into a functional phenotype, which is termed *normalization* according to a related concept in clinical biomodulatory anticancer therapy (Heudobler et al., 2019a, Heudobler et al., 2019b) and immunotherapy (Sanmamed and Chen, 2018; Wang J. et al., 2019). Clinically, ATO alone cures about 70% of APL patients (Ghavamzadeh et al., 2011), while ATRA alone did not achieve long-term remissions (Castaigne et al., 1990), highlighting the necessity of the combination treatment and the beneficial impact of the metalloid. Still, remissions in clinical studies using low dose biomodulatory treatments are explicitly linked to differentiation (Thomas et al., 2015).

APL represents one of several subgroups of acute myeloid leukaemia (AML), which is a heterogenous malignancy due to differences in morphology, chromosomal translocations and mutations in genes responsible for proliferation and differentiation (De Kouchkovsky and Abdul-Hay, 2016). Treatment of AML patients, excluding APL, involves cytotoxic chemotherapy with cytarabine and anthracyclines in combination with stem cell transplantation (Dohner et al., 2010), but this regime is often not tolerated by elderly patients, which represent the main patient population (De Kouchkovsky and Abdul-Hay, 2016). Thus, alternative approaches are being evaluated for AML, including normalization, check-point inhibitors and immunotherapies (Winer and Stone, 2019). Of note, biomodulatory low-dose treatment regimens using transcriptional modifiers have already been successfully tested in the clinical setting (Heudobler et al., 2018b), especially for some forms of AML (Thomas et al., 2015; Heudobler et al., 2018a). Further proof-of-principle studies exist that support the transfer of normalization to other subgroups of AML (El Hajj et al., 2015; Laouedj et al., 2017) and also to non-haematological cancer types (Ishay-Ronen et al., 2019). Differentiation was even shown to activate ROS formation and phagocytosis in AML blasts (Klobuch et al., 2018).

Myeloid differentiation into neutrophils and the concomitant reactivation of physiological apoptosis programs (Lawrence et al., 2018) is accompanied by substantial changes at the levels of the transcriptome and proteome (Paul et al., 2015). In recent years, we (Meier et al., 2017; Meier-Menches et al., 2019; Neuditschko et al., 2021) and others (Hu et al., 2016; Wang Y. et al., 2019; Wang et al., 2020) developed proteomic strategies to comprehensively evaluate metallodrug effects in cancer cells. Proteomic technologies rely on mass spectrometry to analyse the protein fraction of cells after their tryptic digestion into peptides (Bantscheff et al., 2012). The peptides are identified by their accurate mass and their sequence is determined by tandem mass spectrometry. By means of bioinformatic processes, the peptide sequences are aligned to the known protein sequences by means of databases. Several peptide sequences of the same protein are summed up and the corresponding protein intensity is calculated. The calculated protein abundances from the mass spectrometric intensities are then used to compare the protein abundances of all identified proteins of different conditions, *e.g.*, control *vs.* drug treated. Since the clinically used combination treatment consists of an inducer of differentiation (ATRA) and a metalloid (ATO), we focus on elucidating the differentiation– and metal-specific effects in the combination treatment in the APL cell line NB4 (FAB M3) *in vitro*. Furthermore, we extend this approach to cell lines of other AML subgroups, including HL-60 (FAB M2) and U937 (FAB M5). These cell lines correspond to different stages of myeloid differentiation (Tschan et al., 2010; Jensen et al., 2015; Orfali et al., 2015; Sumi et al., 2016). ATRA and ATO-treated AML cancer cell lines were previously investigated (Tschan et al., 2010; Valenzuela et al., 2014; El Hajj et al., 2015; Jensen et al., 2015; Sumi et al., 2016), including transcriptomics and gel-based proteomics approaches (Zheng et al., 2005), yet, shotgun proteomics was not reported so far to the best of our knowledge. The characterization of metal-specific effects of the combination treatment *in vitro* helps understanding the clinically relevant effects of ATO and transferring this insight to establish novel combination treatments for AML, possibly beyond APL.

## Materials and Methods

### Materials

*All*-trans retinoic acid (ATRA), arsenic trioxide (ATO) and phorbol 12-myristate 13-acetate (PMA) were purchased from Sigma-Aldrich and used as received. The compound [chlorido (*η* ^*6*^-p-cymene)(*N*-(4-fluorophenyl)-2-pyridinecarbothioamide)Ru(II)] chloride (plecstatin-1) was prepared according to previously published procedures (Meier et al., 2013, Meier et al., 2017). Dimethylsulfoxide (DMSO) was obtained from Sigma and used as received. AlamarBlue reagent was purchased from Thermo Fisher Scientific.

### Cell Culture

The experiments were performed with the cancer cell lines HL-60 (acute myelocytic leukaemia, FAB M2; cell line ontology CLO:0003775), NB4 (acute promyelocytic leukaemia, FAB M3; cell line ontology CLO:0007947) and U937 (histiocytic lymphoma, FAB M5; cell line ontology CLO:0009465). HL-60 cells were kindly provided by M. Jakupec (Faculty of Chemistry, University of Vienna, Austria) and NB4 cells were a kind gift of M. Tschan (Institute of Pathology, University of Berne, Switzerland). U937 cells were purchased from ATCC. The suspension cells were cultured in RPMI-1640 medium including l-glutamine (Gibco, Life Technologies, United Kingdom). All media contained 10% heat-inactivated fetal calf serum (FCS, ATCC, United States) and 1% penicillin/streptomycin (ATCC, United States). Cells were grown in a humidified atmosphere containing 5% CO_2_ and 95% air at 37°C.

### Viability Assay

The resazurin-based alamarBlue cell viability assay was used to investigate the cytotoxicity of arsenic trioxide (ATO, 2 mM stock in basic aqueous solution) or plecstatin-1 (2 mM stock in DMSO) in combination with *all*-trans retinoic acid (ATRA, 5 mM stock in EtOH). Cells were seeded in densities of 30,000–50,000 cells/well in flat-bottom 96-well plates (Corning) in 200 µl of the respective medium. The HL-60 and NB4 cells were co-treated with ATRA (0.1 µM) and the dilution series of ATO or plecstatin-1. The U937 cells were co-treated with PMA (0.1 µg ml^−1^) and the dilution series of ATO or plecstatin-1. After the incubation time of 48 h, alamarBlue reagent (Invitrogen) was added (10% v/v) to the cells and further incubated for 4 h. Then, the cells were pelleted (500 rpm, 5 min) and the solution was transferred into a black flat-bottom 96-well plate (Corning) and the fluorescence intensity was acquired using a plate reader. Each treatment was blank-corrected and performed in triplicates of triplicates and concentrations for 50% growth inhibition (IC_50_) after 48 h were obtained by sigmoidal fitting using Graph Pad Prism (Version 6).

### Differentiation Status by Flow Cytometry

HL-60 and NB4 cancer cells were treated for 48 h with ATRA (0.1 and 1 µM) using 2 × 10^5^ cells per well in 6-wells. U937 cancer cells were similarly treated with PMA (0.01, 0.1 and 1 µg ml^−1^). Vehicle treated control cells were also plated. After the incubation time, the cells were washed three times with PBS and put on ice. The differentiation status was assessed by labelling with an anti-CD11b antibody (APC clone D12, BD Bioscience) and subsequent evaluation of the CD11b^+^ cell population. Biological triplicates were analysed per condition on a FACS Canto II cytometer (BD Bioscience).

### Cell Cycle Analysis by Flow Cytometry

NB4 cancer cells were treated with ATRA (0.1 and 1 µM) using 2 × 10^5^ cells per well in 6-wells for 48 h. Thereafter, the cells were washed three times with PBS, resuspended and put on ice. Three biological replicates were analysed per condition. The cell cycle distribution of controls and treatment groups were analysed with the BD cycle test Plus DNA kit (BD Bioscience) according to the manufacturer’s protocol. In brief, cells were washed three times and permeabilized. The amount of interfering RNA was reduced using RNase after which propidium iodide was added as a DNA stain and was measured in the PE channel.

### Proteomics

**Treatment**. Cancer cells were typically treated for 48 h at doses corresponding to half IC_50_ concentrations in complete medium using 2×10^6^ cells in T25 flasks. HL-60 cancer cells were treated with freshly dissolved ATRA (0.1 µM), ATRA (0.1 µM) + ATO (0.5 µM) and ATRA (0.1 µM) + plecstatin-1 (5 µM). NB4 cancer cells were treated with freshly dissolved ATRA (0.1 µM), ATRA (0.1 µM) + ATO (0.3 µM) or ATRA (0.1 µM) + plecstatin-1 (3 µM). U937 cancer cells were treated with freshly dissolved PMA (0.1 µg ml^−1^), PMA (0.1 µg ml^−1^) + ATO (0.35 µM) or PMA (0.1 µg ml^−1^) + plecstatin-1 (4.5 µM). See also Supplementary Tables S1,S2. Each condition was analysed in biological triplicates.

**Cellular Fractionation**. HL-60, NB4 and U937 cells were fractionated into cytoplasmic and nuclear extracts as previously described (Kreutz et al., 2017). All steps were performed on ice. Briefly, the cells were extensively washed with PBS (1×). Isotonic lysis buffer (10 mM HEPES, 10 mM NaCl, 3.5 mM MgCl_2_, 1 mM EGTA, 0.25 M Sucrose, 0.5% Triton X-100) containing protease inhibitors (1% PMSF and 1% protease and phosphatase inhibitor cocktail from Roche) was added, the cells were scraped off and transferred into labelled 15 ml Falcon tubes (17 × 120 mm, Corning). The cellular membrane was ruptured using shear stress by pressing the cell suspension through a syringe (23 G). After centrifugation (3,500 rpm, 4°C, 5 min), the supernatant containing the cytoplasmic protein fraction was transferred into ice-cold ethanol (1:5) and precipitated over night at –20°C. The pellet containing the nuclei was incubated with a hypertonic solution (10 mM Tris-HCl, 1 mM EDTA and 0.5 M NaCl) and subsequently with NP-40 buffer (10 mM Tris-HCl, 1 mM EDTA and 0.5% NP-40) containing protease inhibitors (1% PMSF from Sigma and 1% protease and phosphatase inhibitor cocktail from Roche). After centrifugation (3,500 rpm, 4°C, 5 min), the soluble nuclear proteins were transferred into ice-cold ethanol (1:5) and precipitated over night at –20°C. The precipitated proteins were pelleted, dried under vacuum and dissolved in sample buffer (100 mM dithiothreitol (DTT), 7.5 M urea, 1.5 M thiourea, 4% CHAPS, 0.05% sodium dodecyl sulphate) and the protein concentration was determined using the Bradford assay. As an example, U937 cytoplasmic fractions contained roughly 5–10 µg µl^−1^ protein and U937 nuclear fractions roughly 2–4 µg µl^−1^.

**Digestion protocol**. The HL-60, NB4 and U937 samples obtained by the nucleo-cytoplasmic fractionation protocol were digested in-solution as previously described according to the filter-aided sample preparation (FASP) protocol (Kreutz et al., 2017). Equal amounts of 20 µg protein per sample were reduced with DTT at 37°C. They were then loaded on 10 kDa centrifugal filters (Microcon-10, Merck, Millipore) and were preconcentrated. The samples were carbamidomethylated with iodoacetamide in the dark. The samples were digested over night with trypsin/lys-C (Promega, Germany) at 37°C. Filters were washed with 0.5% TFA and the eluates were dried with a miVac duo concentrator (GeneVac Ltd., United Kingdom) and stored at –20°C until analysis.

**LC-MS/MS analysis**. The data was acquired on a QExactive Orbitrap mass spectrometer (Thermo Scientific, Germany), which was coupled with a nanoLC-system (Dionex Ultimate 3,000, Thermo Scientific, Germany). The LC was equipped with a C-18 separation column (Dionex, Acclaim PepMap RSCL, 75 µM × 50 cm) and C-18 trapping column (2 cm × 100 µm). Dried samples were dissolved in formic acid (30%, 5 µl) containing 10 fmol of four synthetic peptides (Glu1-fribrinopeptide B: EGVNDNEEGFFSAR; M28: TTPAVLDSDGSYFLYSK; HK0: VLETKSLYVR and HK1: VLETK (ε-acetyl)SLYVR) and were diluted with mobile phase A (40 µl), which consisted of 98% water, 2% acetonitrile, 0.1% formic acid. Mobile phase B consisted of 20% water, 80% acetonitrile and 0.1% formic acid. Each biological sample was recorded in technical duplicates. Samples were analysed over a 135 min chromatographic run containing a 90 min gradient from 8 to 40% mobile phase B. MS1 resolution was 70 k with 50 ms injection time and MS2 resolution was 17.5 k with 75 ms injection time. A top eight method was used in the mass range of 400–1,400.

**Data analysis**. MaxQuant (Version 1.6.8.0), including the in-built Andromeda search engine, was used for label-free quantification. For identification, we used only non-redundant Swissprot entries with at least two peptides (of which one needed to be unique). The first and main search peptide tolerance was 50 and 25 ppm, respectively. The false discovery rate (FDR) was fixed to 0.01 on the peptide and protein level. The statistical evaluation was performed with Perseus software (Version 1.6.6.0) using LFQ intensities of the MaxQuant result file. After filtering potential contaminants the LFQ values were Log (2)-transformed. Technical duplicates were averaged during data evaluation. Only proteins detected in three of three biological replicates in either control and/or treatment groups were considered for data evaluation. Permutation-based FDR was set to 0.05 for t-tests and gave multi-parameter corrected significant regulations of protein abundance (S0 = 0.1). The final data set was further analysed using web-based applications (*e.g.,* DAVID bioinformatics Resources 6.8).

## Results

**FIGURE 2:**
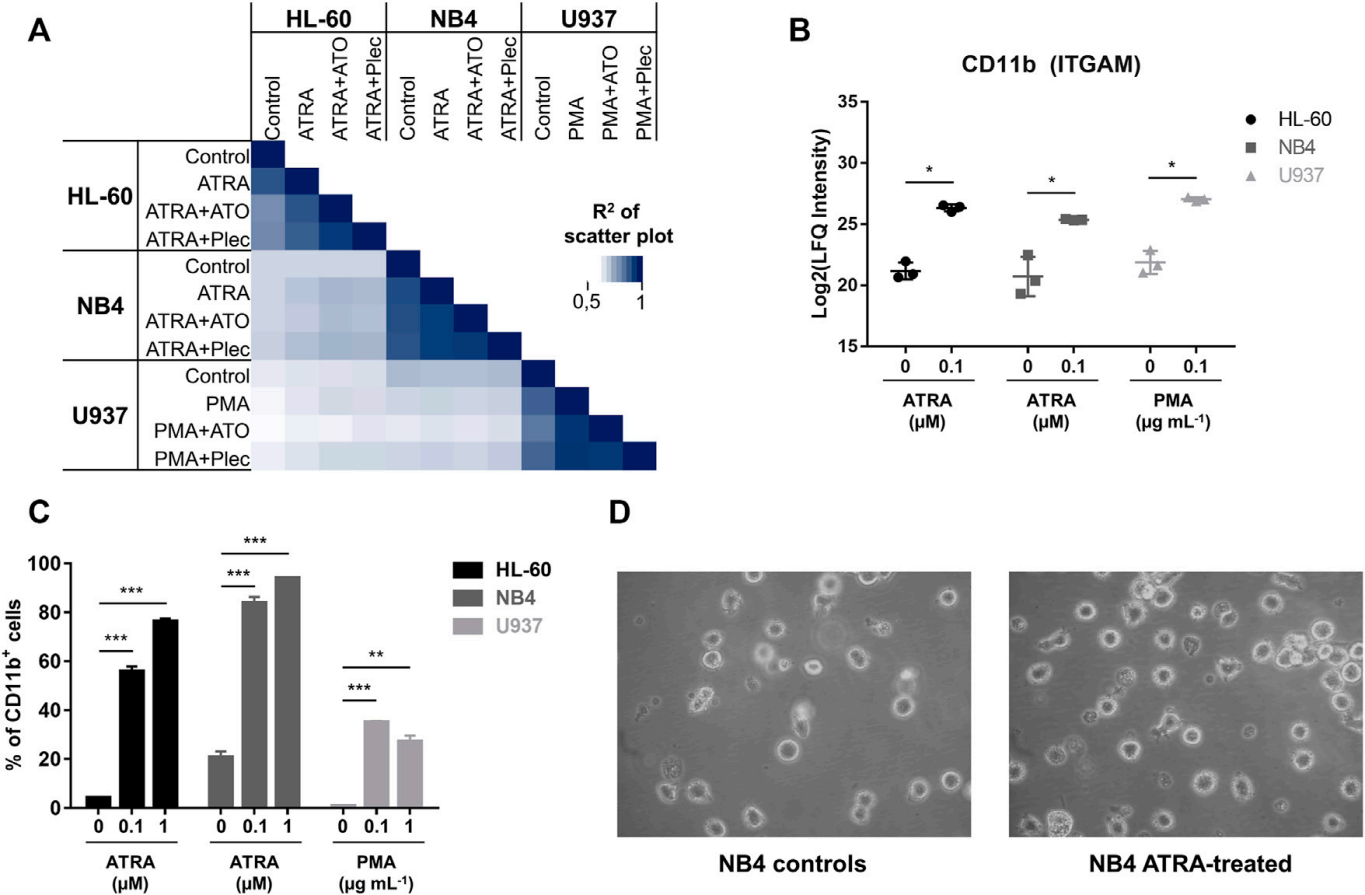
**(A)** Multi-correlation plot displaying the R^2^-correlation among all investigated conditions. The abundances of cytoplasmic proteins were used. Biological triplicates of each condition were averaged to the mean**. (B)** Intracellular protein abundance of CD11b (ITGAM) in the cytoplasmic fraction of the three AML cancer cell lines according to differentiation with either ATRA or PMA for 48 h. Significance: * = multi-parameter corrected significant regulation. **(C)** Flow cytometric analysis of CD11b^+^ cells (surface expression) in all three AML cancer cell lines in dependence of ATRA (PMA) treatment. The cells were differentiated for 48 h. Three biological replicates were analysed. Significance: * *p*-value < 0.05, ** *p*-value < 0.005 and *** *p*-value < 0.0005. **(D)** Representative light microscopy images (Zeiss) of control and ATRA-treated NB4 cells.

All differentiation treatments were carried out with ATRA (0.1 µM) in HL-60 and NB4 cancer cells, and phorbol 12-myristate 13-acetate (PMA, 0.16 µM = 0.1 µg ml^−1^) in U937 cancer cells by incubating for 48 h, in accordance to previous studies (Zheng et al., 2005; Orfali et al., 2015; Sumi et al., 2016). The concentrations for 50% growth inhibition (IC_50_) of the combination treatment were assessed by the alamarBlue assay using always the same concentration of ATRA or PMA (Supplementary Table S1). Sub-cytotoxic concentrations corresponding to half-IC_50_ values of the metal(loid) were used for the subsequent proteome experiments (Supplementary Table S2). Differentiation processes and metal-specific effects were comprehensively evaluated using mass spectrometry (MS)-based shotgun proteomics by a label-free quantification (LFQ) approach (Supplementary Scheme S1), similarly to previous protocols (Kreutz et al., 2017; Meier-Menches et al., 2019). Label-free quantification implies that the proteins or peptides are not labelled with stable isotopes to compare different conditions (Bantscheff et al., 2012). Accordingly, the samples are separately processed, digested and analysed. They are only combined during the bioinformatic evaluation. Therefore, LFQ proteomics requires a robust workflow since every step of sample preparation and analysis may introduce bias, but offers a facile comparison of many samples. We perpared controls, differentiation induction and co-treatments in the three AML cell lines HL-60, NB4 and U937 (Figure 2A). The AML cancer cells were fractionated into cytoplasmic (CYT) and nuclear extract (NE) fractions. Aliquots of 20 µg protein of each fraction were digested into peptides by a filter-assisted proteolytic digestion using trypsin/Lys-c. The obtained peptide samples were individually analysed by nano-liquid-chromatography MS (nLC-MS) using a 90 min gradient. A total of 5′524 proteins were identified in the 144 individual nLC-MS runs by applying an FDR of 0.01 on the protein and peptide level and by merging protein isoforms. The protein abundances of the (co-)treatments revealed strong correlations among conditions of the same cancer cell line, while correlations among cancer cell lines were less pronounced (Figure 2A). The same trend was also reflected in the principal component analysis (PCA) (Supplementary Figure S1).

### Molecular Characterisation of Differentiation

The three AML cell lines were treated with a differentiation inducer (ATRA or PMA) in order to molecularly characterize differentiation processes. The differentiated state is then further used to subtract these effects from the combination treatment to obtain metal-specific effects in the following subchapters.

In our treatment panel, ATRA–and PMA-induced differentiation revealed significant expression changes on the order of 10% of the respective proteomes (Supplementary Figure S2A). All three treated AML cell lines featured common significantly enriched proteins of the KEGG pathway “leukocyte transendothelial migration” (corrected *p*-value <0.05) (Schimmel et al., 2017). Of the 22 significantly up-regulated proteins in the three differentiated AML cancer cell lines in the cytoplasmic fraction (CYT), a subset of 14 proteins was directly related to differentiation and immune processes, including canonical markers of myeloid differentiation (Supplementary Figures S2B,C). These correspond to proteins influencing cell adhesion, for example integrin-αM (ITGAM, CD11b), integrin β2 (ITGB2, CD18), intercellular adhesion protein 1 (ICAM1) and the protein-tyrosine kinase 2β (PTK2B). Second, proteins involved in immune responses were up-regulated, namely protein S100-A9 (S100A9), neutrophil cytosolic factor 1 (NCF1), tyrosine-protein phosphatase non-receptor type substrate 1 (SIRPA) and tyrosine-protein phosphatase non-receptor type 6 (PTPN6). Third, up-regulation of long-chain-fatty-acid–CoA ligase 1 (ACSL1), pyruvate kinase PKM (PKM), 6-phosphogluconate dehydrogenase (PGD) and arachidonate 5-lipoxygenase-activating protein (ALOXP5AP) indicated metabolic remodelling in these cells accompanying differentiation. A significant induction of CD11b was observed in the CYT fraction of HL-60 (36-fold), NB4 (25-fold) and U937 (36-fold) (Figure 2B). CD11b is among the main markers for myeloid differentiation. The same trend was independently confirmed by analysing CD11b as a cell surface marker by flow cytometry (Figure 2C and Supplementary Figure S3A). Although the basal CD11b-levels varied slightly among the cancer cell lines, differentiation led to a significant and dose-dependent increase of CD11b^+^ cells. NB4 was found to be the most responsive cell line in absolute cell numbers yielding >80% CD11b^+^ cells, when dosing with 0.1 µM ATRA. Representative images from light microscopy of NB4 controls and ATRA-treated cells are shown in Figure 2D. AML cancer cells are suspension cells and differentiation led in all cases to an enhanced adherent phenotype.

**FIGURE 3:**
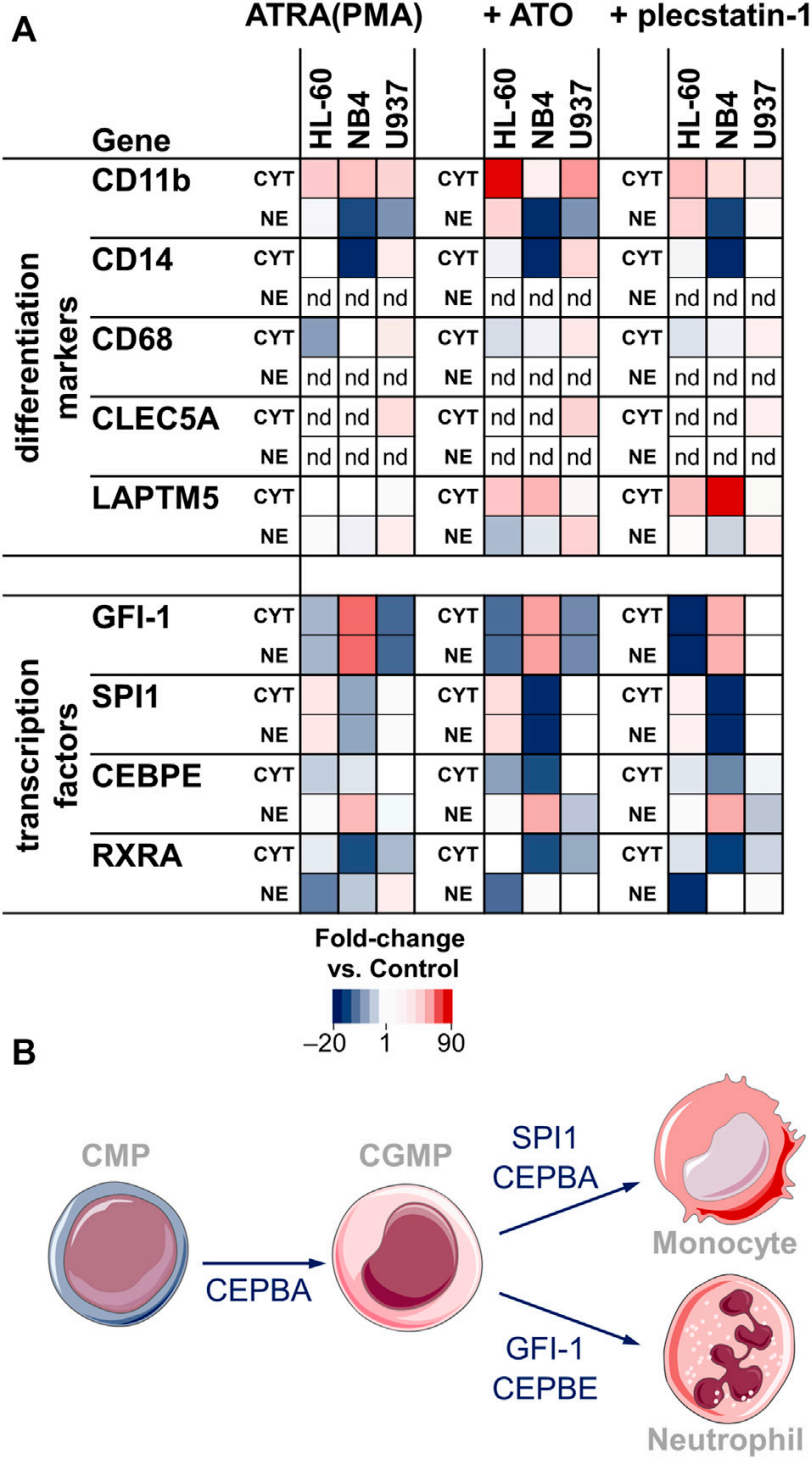
**(A)** Heat map showing the fold-changes of surface marker and transcription factor abundance upon treatment with ATRA (PMA) only (*left*), ATO + ATRA (PMA) (*middle*) and plecstatin-1+ATRA (PMA) (*right*) with respect to controls. Treatments were performed over 48 h. The LFQ intensity of averaged biological replicates was used to create the heat maps. Fold-changes are given as de-logarithmised ratios with respect to untreated controls. CYT = cytoplasmic fraction, NE = nuclear extract fraction, nd = not detected. **(B)** Simplified differentiation cascade of the common myeloid progenitor (CMP) into monocytes and neutrophils through the common granulocyte monocyte progenitor (CGMP). The involved transcription factors are given next to the arrows.

Despite these commonalities, the overlap of the significantly regulated proteins upon differentiation was low (Supplementary Figure S2B). This is also reflected by the distinct regulation of other lineage markers and transcription factors (Figure 3A, *left*). The monocyte-macrophage markers CD14 and CD68 were detected in CYT fractions of all 3 cell lines, but found up-regulated only in U937 cells, corresponding to their monocytic character. Interestingly, CD14 was down-regulated in NB4 while CD68 was down-regulated in HL-60. Additionally, the macrophage marker c-type lectin domain family five member A (CLEC5A) was only detected in U937 cells.

Some of the main transcription factors that regulate myeloblast/promyelocyte differentiation into neutrophils/macrophages were detected in our data set (Figures 3A,B). The zinc finger protein GFI-1 is a transcription factor that promotes granulocytic development and inhibits proliferation in myeloid progenitors. GFI-1 was found up-regulated in NB4 and down-regulated in HL-60 and U937 cells. Accordingly, differentiation led to a significant increase in the G0/G1 fraction of NB4 cells (Supplementary Figure S3B). The transcription factors SPI1 (PU.1) and CCAAT/enhancer-binding protein-α (CEBPA) direct macrophage development. SPI1 showed an inverse trend compared to GFI-1. It was down-regulated in NB4, up-regulated in HL-60 and constant in U937. CEBPA was not significantly regulated upon differentiation. However, it was highly abundant in the nuclear extract (NE) fraction in U937, followed by HL-60, while NB4 displayed the lowest abundance of CEBPA (Supplementary Figure S2D). This indicates that the basal levels of CEBPA are not significantly affected by ATRA or PMA treatments. In contrast to CEBPA, CCAAT/enhancer-binding protein-ε (CEBPE) is a transcriptional activator that controls the promyelocyte-to-myelocyte transition in granulocytes. In NB4 cells, the down-regulation of CEBPE in the cytoplasmic fraction and concomitant upregulation in the nuclear fraction seems to indicate a translocation event into the nucleus and may emphasize their susceptibility to ATRA-induced differentiation along the granulocytic lineage.

Together, this data indicates the successful induction of differentiation in AML cancer cells with ATRA (PMA) within the 48 h incubation time and supports the feasibility of this approach. The regulations of the differentiation markers and transcription factors underline characteristic differentiation processes of the AML subgroups along granulocytic and monocytic/macrophage lineages.

### ATO-Specific Effects in the Combination Treatment

ATO-specific effects were obtained by subtracting the effects of the differentiation inducer in each cell line from the effect of the combination treatment.

**Differentiation**. Differentiation was only subtly affected by the combined treatment compared to ATRA/PMA alone (Figure 3A). In HL-60 cancer cells, CD11b seemed to be more strongly upregulated in the combination treatment. The transcription factors were not markedly regulated by ATO-specific effects. In NB4 cancer cells, the combination treatment led to a stronger down-regulation of SPI1 in the cytoplasmic and nuclear fractions and a more pronounced down-regulation of CEBPE in the cytoplasmic fraction.

**FIGURE 4:**
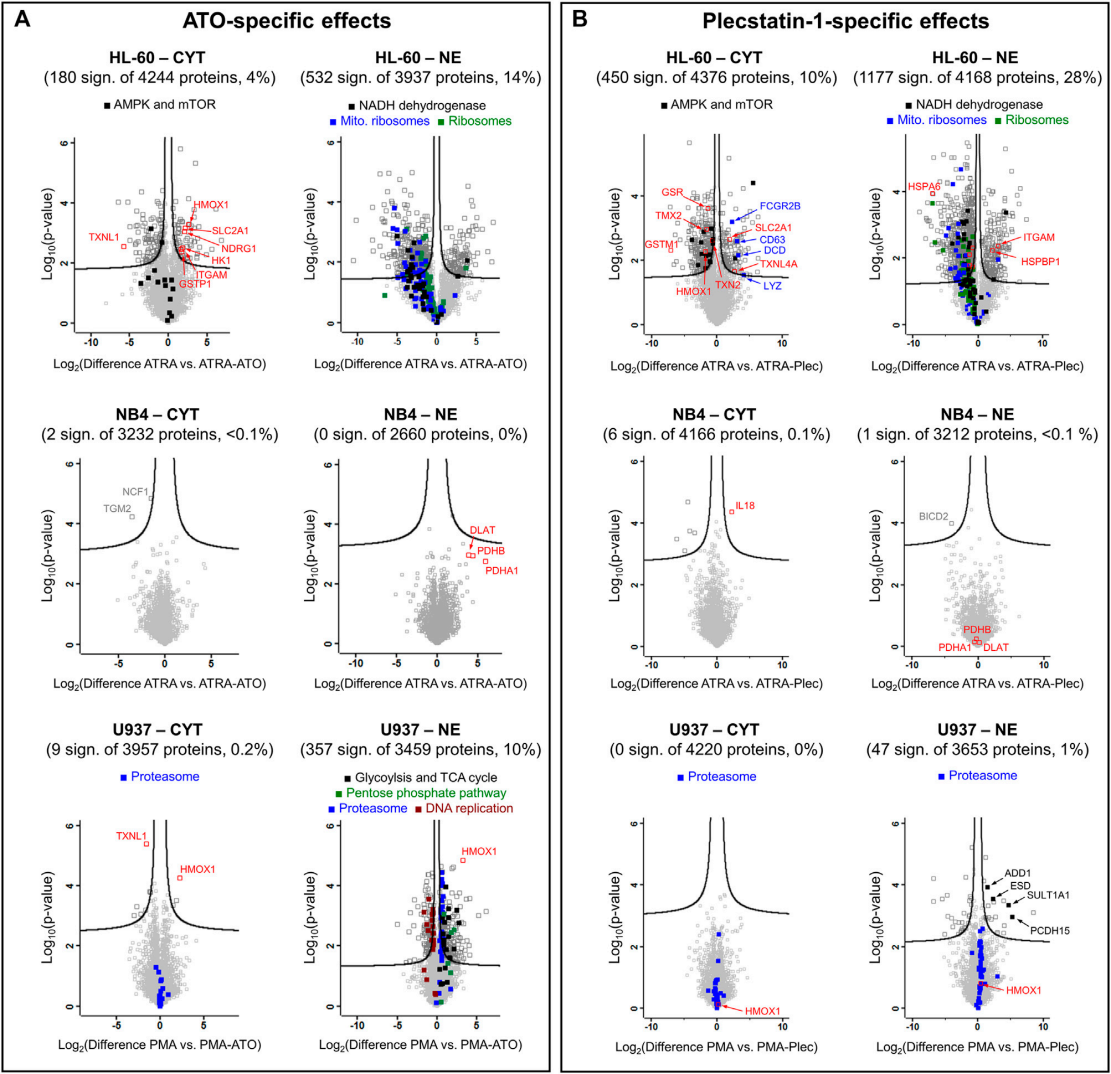
Volcano plots of HL-60, NB4 and U937 cancer cell lines treated with **(A)** ATO + ATRA (PMA) or **(B)** plecstatin-1+ATRA (PMA) over 48 h in comparison to differentiation with ATRA (PMA) alone. The Log_2_(differences) refer to logarithmised differences of LFQ intensity of a given protein between the two conditions. Multi-parameter corrected significances were calculated with Perseus (Version 1.6.6.) using an FDR = 0.05 and S0 = 0.1. Proteins are represented by squares. Significantly regulated proteins are given in dark grey, non-regulated proteins in light grey.

**HL-60 cells**. Despite these similarities, the ATO + ATRA (PMA) combination triggered distinct cellular responses in HL-60 cells compared to differentiation alone (Figure 4A) and showed proteome alterations in the cytoplasmic (4%) and nuclear (14%) fractions highlighting that ATO-specific effects are pronounced. Many of these effects were linked to cytoprotective responses. The upregulation of protein NDRG1 (Supplementary Figure S2D), heme oxygenase 1 (HMOX1) and glutathione S-transferase P (GSTP1) indicated oxidative stress. Interestingly, ATO + ATRA treatment also up-regulated the facilitated glucose transporter member 1 (SLC2A1) and hexokinase-1 (HK1) that suggest an increased energy demand of these cells. The metal-specific effects of the co-treatment in the nuclear fraction of HL-60 cells included global down-regulation of cytoplasmic and mitochondrial ribosomes, as well as of the NADH dehydrogenase complex as a result of a prolonged integrated stress response (ISR), which is a known feature of ATO (Figure 4A).

**NB4 cells**. ATO in the co-treatment did not significantly affect the proteome of NB4 cells compared to differentiation only (Figure 4A). The clinical success of the ATO + ATRA treatment over ATRA alone seems to be associated with a reduced proteomic plasticity of the NB4 cancer cells, *i.e.*, by a reduced capability of these APL cancer cells to cope with the additional impact of the metalloid. Pyruvate dehydrogenase components (DLAT, PDHA1 and PDHB) were uniformly upregulated in the nuclear fraction.

**U937 cells**. U937 cells showed few protein regulations upon combined ATO + PMA treatment over PMA treatment alone in the cytoplasmic fraction, while a more pronounced proteomic response was observed in the NE fraction (10%) (Figure 4A). HMOX1 and TXNL1 were significantly up- and down-regulated, respectively, in the cytoplasmic fraction in analogy to treated HL-60 cells. HMOX1 was also up-regulated in the nuclear fraction. Furthermore, proteins of the proteasome complex and carbon metabolism were uniformly upregulated in the nuclear fraction of U937, the latter including glycolysis, tricarboxylic acid cycle and pentose phosphate pathway and again represent specific cytoprotective responses of U937 cells against ATO-induced stress. Proteasomal proteins were only upregulated in the NE fraction, while the cytoplasmic fraction did not show such an effect (Figure 4A). In contrast, proteins involved in DNA replication were uniformly down-regulated suggesting reduced proliferative capacity, as expected of the maturation of these cells into monocytes/macrophages.

### Plecstatin-1-Specific Effects in the Combination Treatment

In general, plecstatin-1 was about 10-fold less potent than ATO in the tested AML cell lines. Similar to ATO, the plecstatin-1-specific effects were obtained by subtracting the effects of differentiation induction with ATRA or PMA from the ones of the combination treatments (Figure 4B).

**Differentiation**. The effect of plecstatin-1 in the combination treatment on differentiation was only minor (Figure 3A). Although the down-regulation of GFI-1 seemed to be more pronounced in HL-60 cells, plecstatin-1 did not affect GFI-1 abundance in U937 cells. Differentiation markers and transcription factors were generally unaffected in U937 cells. Interestingly, lysosomal-associated transmembrane protein 5 (LAPTM5) was preferentially induced upon co-treatment and strongly induced upon plecstatin-1 co-treatment in NB4 cells. LAPTM5 is expressed in adult hematopoietic cells and serves as an additional differentiation marker (Adra et al., 1996).

**HL-60 cells**. Plecstatin-1 had pronounced effects on the proteome of HL-60 cells in both fractions in a very similar manner to ATO (Figure 4B). In the HL-60 nuclear fraction, the co-treatment with plecstatin-1 significantly increased the expression of CD11β (ITGAM). The global down-regulation of cytoplasmic and mitochondrial ribosomes, as well as proteins of oxidative phosphorylation (*e.g.*, NADH dehydrogenase) indicate induction of an ISR in analogy to ATO. Moreover, the associated down-regulation of ribosomal protein S6 kinase (RPS6KB1) may be responsible for reduced activity of cytoplasmic ribosomes, which were globally down-regulated during this sub-cytotoxic treatment. In parallel to the ATO + ATRA treatment SLC2A1 was also up-regulated by plecstatin-1. In contrast to ATO, a down-regulation of HMOX1 and glutathione-producing proteins was observed (*e.g.*, GSR or GSTM1). Moreover, plecstatin-1+ATRA treatment led to an additional uniform down-regulation of AMP-activated protein kinases (AMPKs) and mTOR signalling, which was not observed for ATO + ATRA (Figure 4A).

**NB4 cells.** The co-treatment using plecstatin-1+ATRA caused only marginal changes in NB4 cells compared to differentiation alone (Figure 4B). Again, this is supposed to be due to a reduced plasticity of the APL cells to mount cytoprotective responses against metal-specific stress. In contrast, plecstatin-1 induced interleukin-18 (IL18) significantly, which is a component of the inflammasome (Van de Veerdonk et al., 2011). Yet, the expression of IL18 is higher in U937 compared to NB4, while HL-60 cancer cells featured medium values (Supplementary Figure S4).

**U937 cells**. No significant protein regulations were observed when treating U937 cancer cells with plecstatin-1+PMA in the cytoplasmic fraction compared to differentiation alone, while some protein regulations were observed in the nuclear fraction. Consequently, the plecstatin-1+PMA co-treatment did not invoke comparable effects to the ATO + PMA combination treatment (Figure 4B). The plecstatin-1+PMA combination generated a minor heterogenous response. The upregulation of sulfotransferase 1A1 (SULT1A1) and S-formylglutathione hydrolase (ESD) indicated detoxification attempts, while similarly, upregulated protocadherin-15 (PCDH15) and alpha-adducin (ADD1) suggested alteration in cell adhesion and cytoskeleton with a possible impact on migration. It seems that the U937 cells also feature a reduced plasticity to cope with the combination treatment including plecstatin-1.

## Discussion

The combination treatment using ATO + ATRA overcomes the differentiation blockade in APL by efficiently degrading the PML–RARα fusion protein and by forming PML-nuclear bodies (Chen et al., 2011; Lo-Coco et al., 2013; de Thé, 2018). As a result, the leukemic promyelocytes develop into non-cancerous neutrophils, despite the perseverance of leukaemia-specific mutations (Tilly et al., 1986) and this cures >90% of APL patients (Lo-Coco et al., 2013). The profound reprogramming of cancer cells mediated by this normalization therapy represents a promising, but under-investigated alternative to cytotoxic chemotherapy (Heudobler et al., 2019b). This study presents an experimental framework to investigate the molecular basis of such normalization processes in the context of AML *in vitro* using proteomic techniques. Specifically, we were able to extract detailed information about differentiation and ATO-specific effects. These guided us to propose a novel combination treatment based on the implicated pathways.

AML cancer cells of different subgroups were used, including the APL cell line NB4 (FAB M3) and the AML cell lines HL-60 (FAB M2) and U937 (FAB M5). HL-60 and NB4 cancer cells are capable of differentiating along the granulocytic path into neutrophils and require ATRA (Lawrence et al., 2018), while U937 cells differentiate along the monocytic/macrophage lineage and require PMA as differentiation inducer (Figure 3B). The presently explored panel of AML cell lines helps distilling a comprehensive reference profile of cellular responses to the clinically employed combination treatment with ATO + ATRA.

ATRA is known to regulate the expression of many genes (Balmer and Blomhoff, 2002) and indeed, a substantial percentage of the proteome was significantly regulated upon differentiation (Supplementary Figure S2). All differentiated cells expressed a protein signature related to transendothelial migration, which is a crucial process to release the matured neutrophils from the bone marrow into the circulation (Lawrence et al., 2018). Successful differentiation was evidenced by the significant upregulation of the differentiation marker ITGAM (CD11b) as demonstrated via both proteomics and flow cytometry (Figure 2). ITGAM forms a heterodimer with ITGB2 and the resulting integrin-αMβ2 is responsible for adhesion and migration of leukocytes and can also associate with ICAM1 (Solovjov et al., 2005). Upon differentiation the AML suspension cells featured also phenotypically an increased adherence, which corroborated this finding. The differentiation of the myeloblasts/promyelocytes into neutrophils/macrophages follows a tightly regulated sequence of transcription factor activity, involving GFI-1, SPI1, CEBPA and CEBPE (Figure 3) (Lawrence et al., 2018). NB4 cells displayed clearly an ATRA-induced differentiation into myelocytes, while U937 showed PMA-induced differentiation along the monocyte-macrophage lineage. In our hands, HL-60 cells featured a more ambivalent behaviour with a potential to differentiate along both granulocytic (up-regulation of CD11b) and monocytic lineages (up-regulation of SPI1 and down-regulation of GFI-1).

The fact that ATRA alone did not lead to long-term remissions in APL patients (Castaigne et al., 1990) emphasizes the importance of metal-specific effects in the combination treatment for clinical success (de Thé, 2018). Interestingly, the transcription factor signature was not significantly altered when comparing differentiation to the combination treatment suggesting that the impact of the metal (-loid) on differentiation was low and ATO rather affected other pathways. It turned out that these pathways were mainly related to inducible cytoprotective mechanisms. In this respect, the HL-60 cell line featured a cytoprotective ISR signature and response to ROS. Besides targeting PML, ATO is known to effectively induce ROS thereby affecting mito-nuclear communication (Quirós et al., 2016) and multiple signalling pathways (Miller et al., 2002). The former was observed by the upregulated HMOX1, which we also found upregulated in colon carcinoma cells treated with ATO (Kreutz et al., 2017). A prolonged ISR leads to the down-regulation of translational and mitochondrial activity (Quirós et al., 2016), as evidenced by the down-regulation of mitochondrial and cytoplasmic ribosomal proteins, as well as mitochondrial NADH dehydrogenase components (Figure 4A). In contrast, the NB4 cell line featured a reduced plasticity to mount a cytoprotective defence against the combination treatment. The upregulation of pyruvate dehydrogenase components indicated that ATO induced mainly metabolic alterations in this APL cancer cell line. Pyruvate dehydrogenase was previously suggested as a direct target of ATO (Miller et al., 2002). Third, the monocytic U937 cell line featured again the induction of specific cytoprotective mechanisms upon the combination treatment. U937 cells induced carbon metabolism and proteasome expression in the nucleus upon the combination treatment. An increased nuclear proteasome expression was previously reported upon ROS-induced stress in U937 (Ullrich et al., 2000). Moreover, Yan et al. (2007) observed a synergistic effect on apoptosis induction in ATO-treated primary AML cancer cells in combination with the proteasome inhibitor bortezomib. In the *in vitro* model presented here the clinical success of the ATO + ATRA combination treatment seems to be characterized by a lack of plasticity of the APL cells to mount cytoprotective defence mechanisms, which were observed in the non-APL cell lines HL-60 and U937.

Interestingly, the organoruthenium-based plecstatin-1 was recently found to be a ROS-inducer with concomitant activation of a mitochondrial ISR in colon carcinoma cells (Meier et al., 2017; Meier-Menches et al., 2019). Specifically, ROS-induction and ISR are also two distinct features of ATO. However, plecstatin-1 targets the scaffold protein plectin and is believed to be an indirect ROS-inducer because the interaction of plecstatin-1 with plectin affects cytoskeletal organisation in cancer cells and impacts on mitochondrial distribution (Meier et al., 2017). Although featuring a different molecular target compared to ATO, we speculated that plecstatin-1 may be a suitable candidate for normalization therapy in AML in combination with inducers of differentiation, due to the overlapping ROS-generation and ISR induction, *i.e.*, overlapping stress responses in cancer cells that rely on phosphorylation of eIF2α (Wernitznig et al., 2020). Indeed, the response profiles of ruthenium-based plecstatin-1 and ATO in combination with differentiation in this study showed a striking similarity in the affected pathways, especially for AML with granulocytic maturation, *e.g.*, HL-60 and NB4, and suggests that the combination of plecstatin-1 with inducers of differentiation shows promise as a novel combination treatment in AML. In these cell lines, a strong similarity in the extent of the proteomic responses can be observed when comparing the volcano plots of the ATO and plecstatin-1 combination treatments against differentiation treatments alone (Figure 4 and Supplementary Figure S5). This strongly suggests that the overlapping effects of ATO and plecstatin-1 with regard to cytoprotective mechanisms are mediated by their common induction of ROS and an ISR that manifest in a reduction in oxidative phosphorylation and ribosomal expression. In plecstatin-1+ATRA treated HL-60 cells, this seems to be further connected to a reduced AMPK and mTOR signalling. However, pyruvate dehydrogenase proteins were not affected in the plecstatin-1+ATRA treatment indicating that these may be specifically targeted by ATO. Finally, the monocytic U937 cell line featured only a reduced plasticity to mount a cytoprotective response against the plecstatin-1 co-treatment. The characteristic change of the ATO + PMA treatment including proteasome, glycolysis, pentose phosphate pathway and DNA replication were not as pronounced, or even absent when treating with the plecstatin-1 combination. This may be related to the fact that the mode of action of plecstatin-1 includes rather cytoskeletal effects with less impact on metabolism compared to ATO. Consequently, the combination treatment involving plecstatin-1 seems promising to treat APL and monocytic AML.

In summary, this study established an *in vitro* framework to comprehensively characterize molecular effects of combination treatments in AML consisting of an inducer of differentiation and a metal(loid). Interestingly, the clinically used combination treatment consisting of ATO + ATRA to cure APL did not show ATO-specific effects in the APL cell line NB4 *in vitro*, which was attributed to a lack of plasticity of the cancer cells to mount a cytoprotective response. The cytoprotective plasticity was identified as a crucial proxy in this context and a deficient plasticity of cancer cells towards the combination treatment is proposed to be indicative of a successful intervention. Under this hypothesis, the combination treatment including the organoruthenium compound plecstatin-1, showed promising activity against APL (FAB M3) and monocytic AML (FAB M5). This model-based approach shows promise to discover novel combination treatments, although a generalization of this concept requires further verification. This is planned for the near future.

## Data Availability

The dataset generated for this study can be found in the PRIDE (ProteomeXchange, http://proteomecentral.proteomexchange.org/cgi/GetDataset) repository with dataset identifier PXD019619.
