# Supplementary material for: A Proteomic Platform Enables to Test for AML Normalization In Vitro
Source: Front Chem. 2022 Feb 1;10:826346. doi: 10.3389/fchem.2022.826346 (PMC8844467; doi:10.3389/fchem.2022.826346)
Supplement: Supplementary file 1 [file DataSheet1.docx]

Supplementary Material

# Supplementary Figures and Tables

## Supplementary Figures


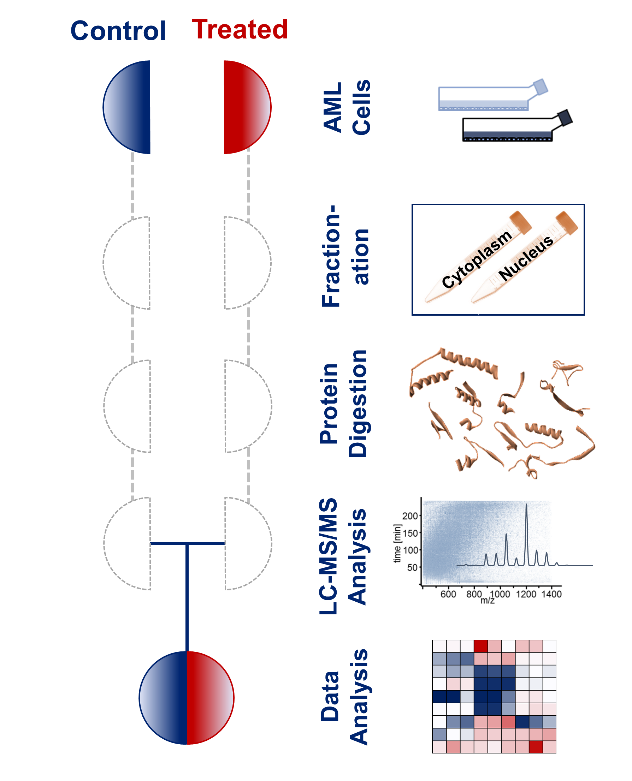


**Supplementary Scheme 1**. Workflow of the response profiling experiments to assess the effect of differentiation and additive/synergistic effects of the metal(-loid) co-treatment on the AML cancer cell lines.

**
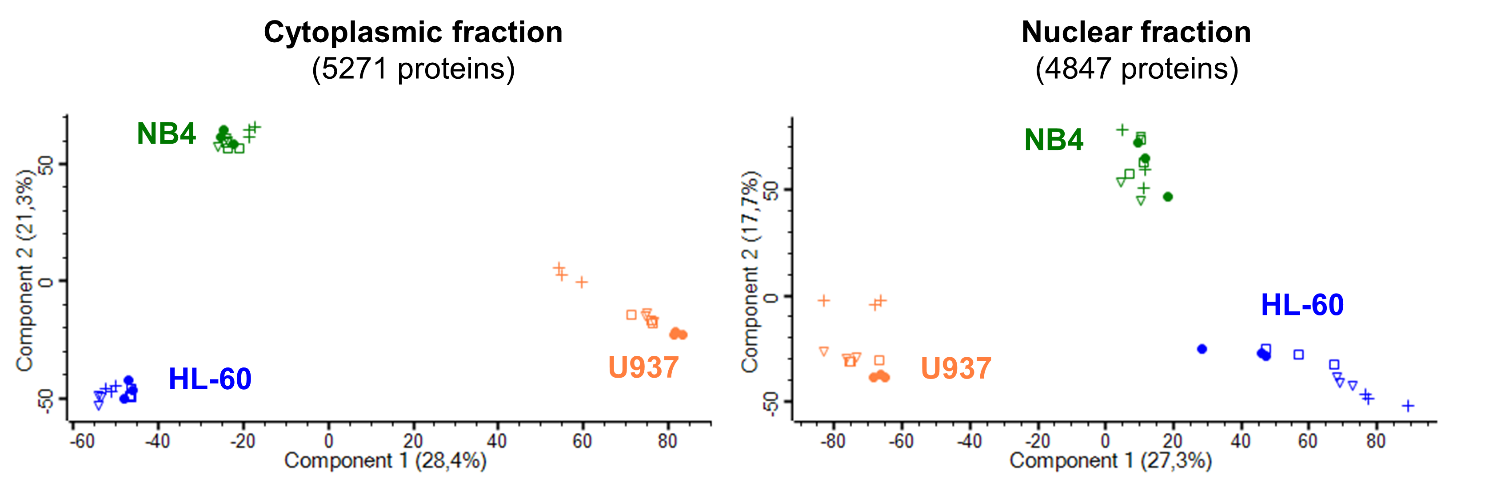
**

**Supplementary Figure 1.** Principal component analysis (PCA) of the employed AML cancer cell lines involving all treatment conditions. The cells were processed according to nuclear-cytoplasmic fractionation. The PCA of the cytoplasmic fraction is shown on the left and the PCA of the nuclear fraction is shown on the right. Samples of control cells of each cell line are indicated with crosses, whereas the samples of treated cells are indicated as follows: ATRA/PMA differentiation only (triangles), ATRA/PMA+ATO (filled circles) and ATRA/PMA+plecstatin-1 (squares).


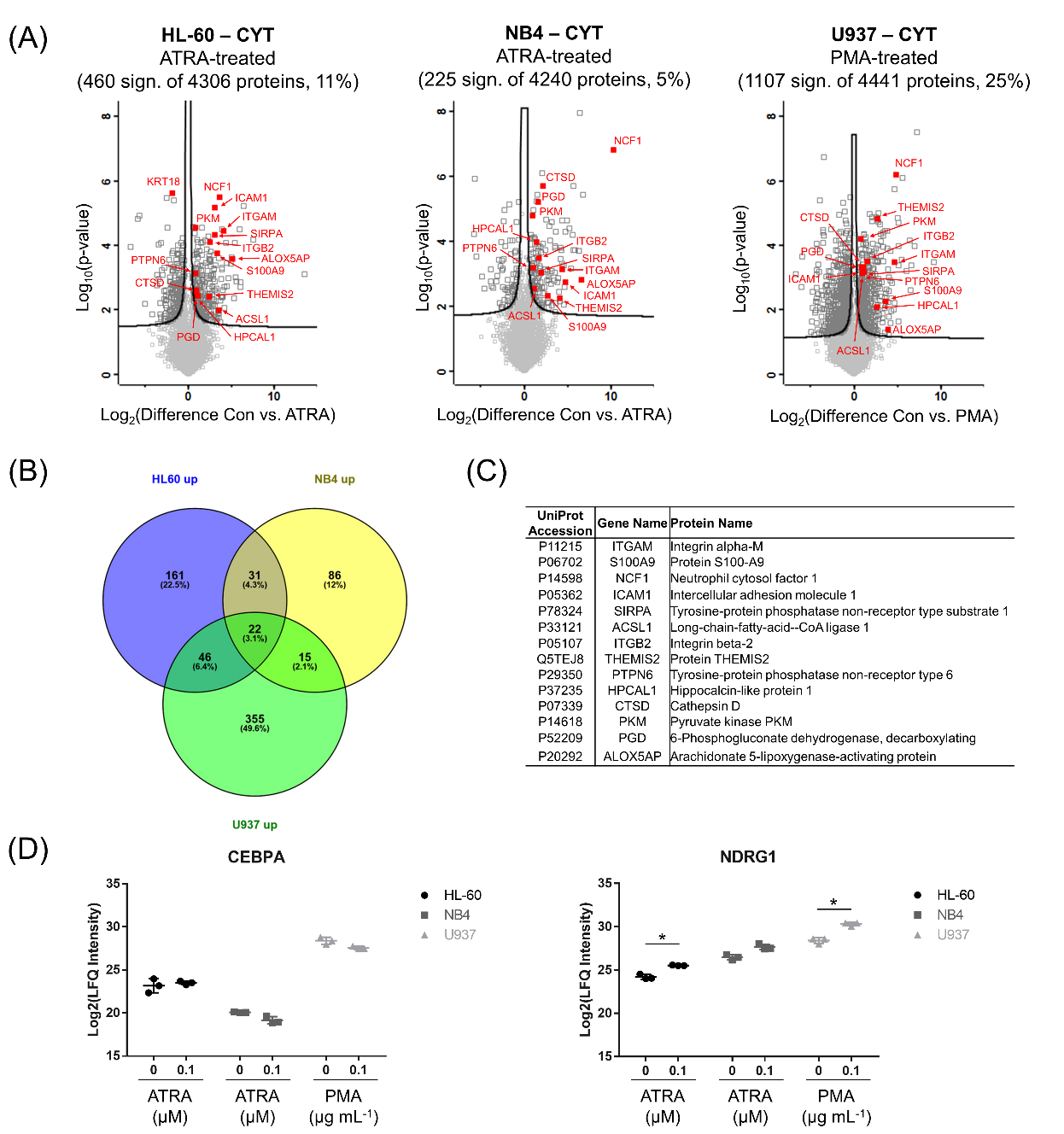


**Supplementary Figure 2.** (**A**) Volcano plots of the three AML cancer cell lines differentiated with ATRA (0.1 µM) or PMA (0.16 µM) over 48 h. The filled red squares denote proteins commonly up-regulated in all three cell lines. HL-60 and NB4 were calculated with an FDR = 0.05 and S0 = 0.1. U937 was calculated with an FDR = 0.01 and S0 = 0.1. Only CYT proteins were showed. (**B**) Venn diagram of the three ATRA or PMA-differentiated AML cancer cell lines showing the overlap of the significantly up-regulated proteins. (**C**) Of the 22 up-regulated proteins common to the three differentiated AML cancer cell lines, 14 are directly related to differentiation and immune processes. (**D**) Protein abundance of CEBPA and NDRG1 in the three AML cancer cell lines according to differentiation with either ATRA or PMA for 48 h. Significance: * p-value < 0.05.


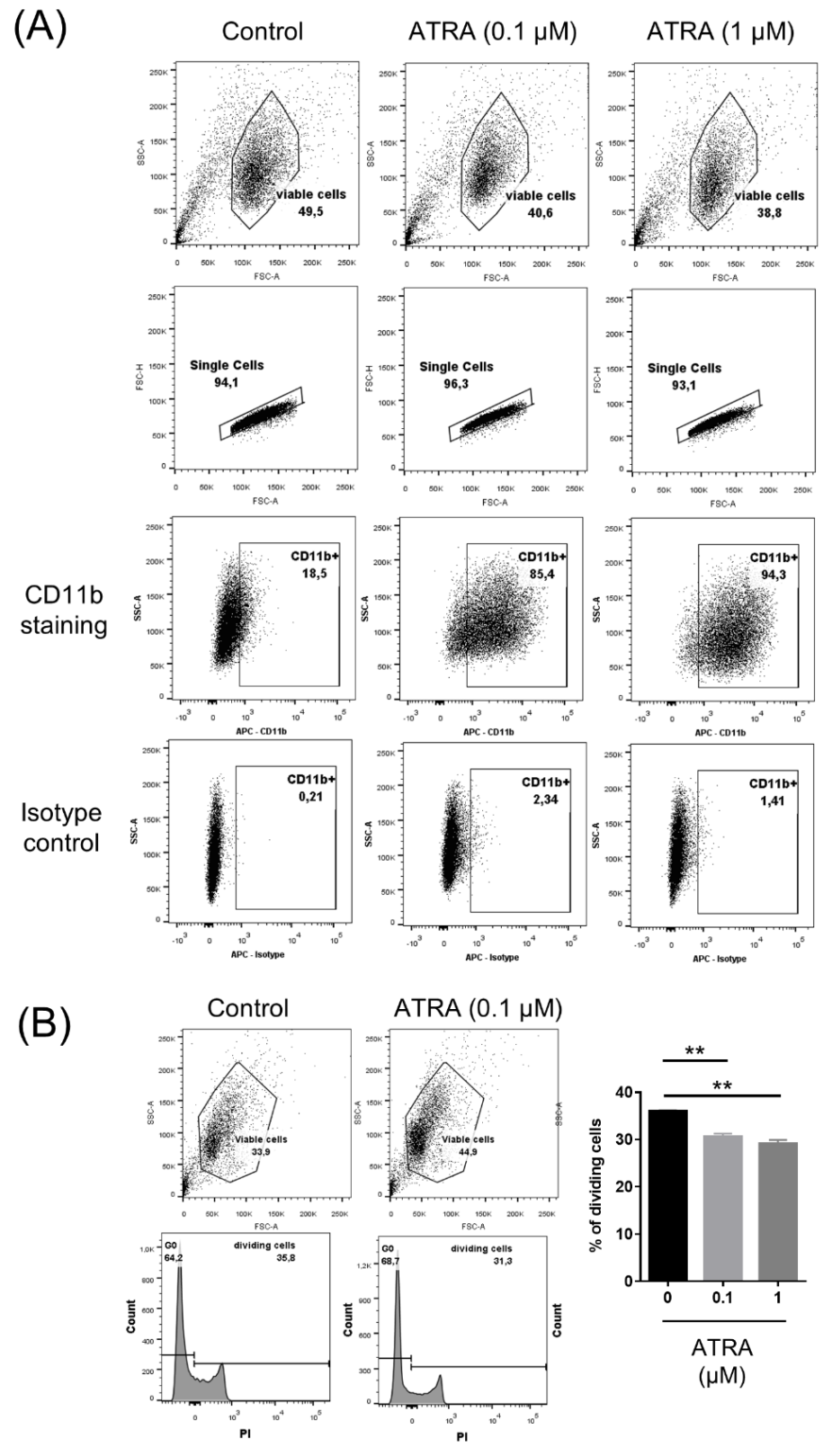


**Supplementary Figure 3.** (**A**) Gating conditions of the flow cytometric analysis of NB4 cancer cells treated with ATRA for 48 h. Cells were stained for CD11b and the CD11b^+^ cell population was determined. The isotype control is given. (**B**) NB4 cancer cells were treated with ATRA for 48 h and the cell cycle distribution was assessed by FACS. ATRA-induced differentiation led to a significant reduction of dividing cells. Significance: ** p-value < 0.005.


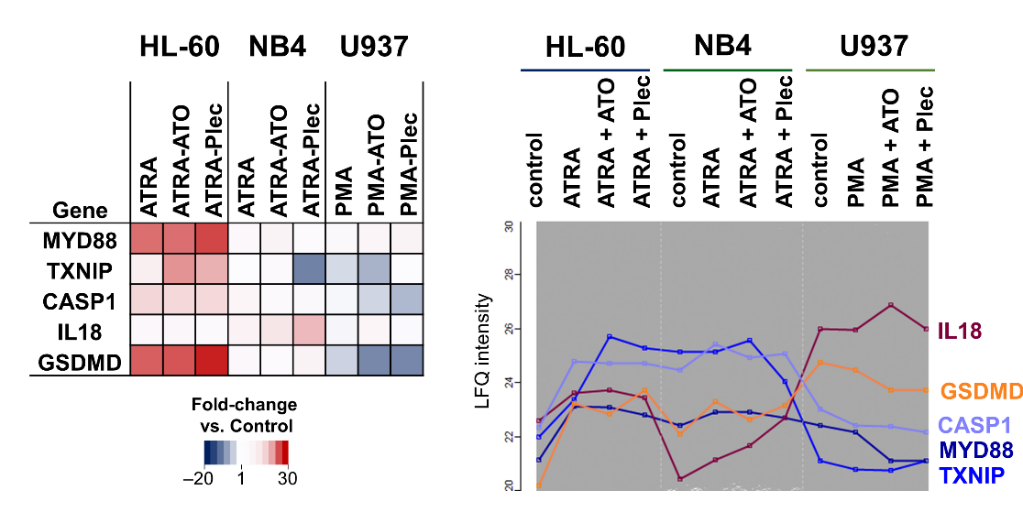


**Supplementary Figure 4.** Regulations of proteins involved in inflammasome signalling, expressed as fold-changes with respect to control cells (*left*). Fold-changes were calculated with averaged LFQ-intensities from the proteome data sets. The LFQ-intensity of each of the components across the different conditions and AML cancer cell lines is shown (*right*). LFQ-intensities of technical and biological replicates were averaged.


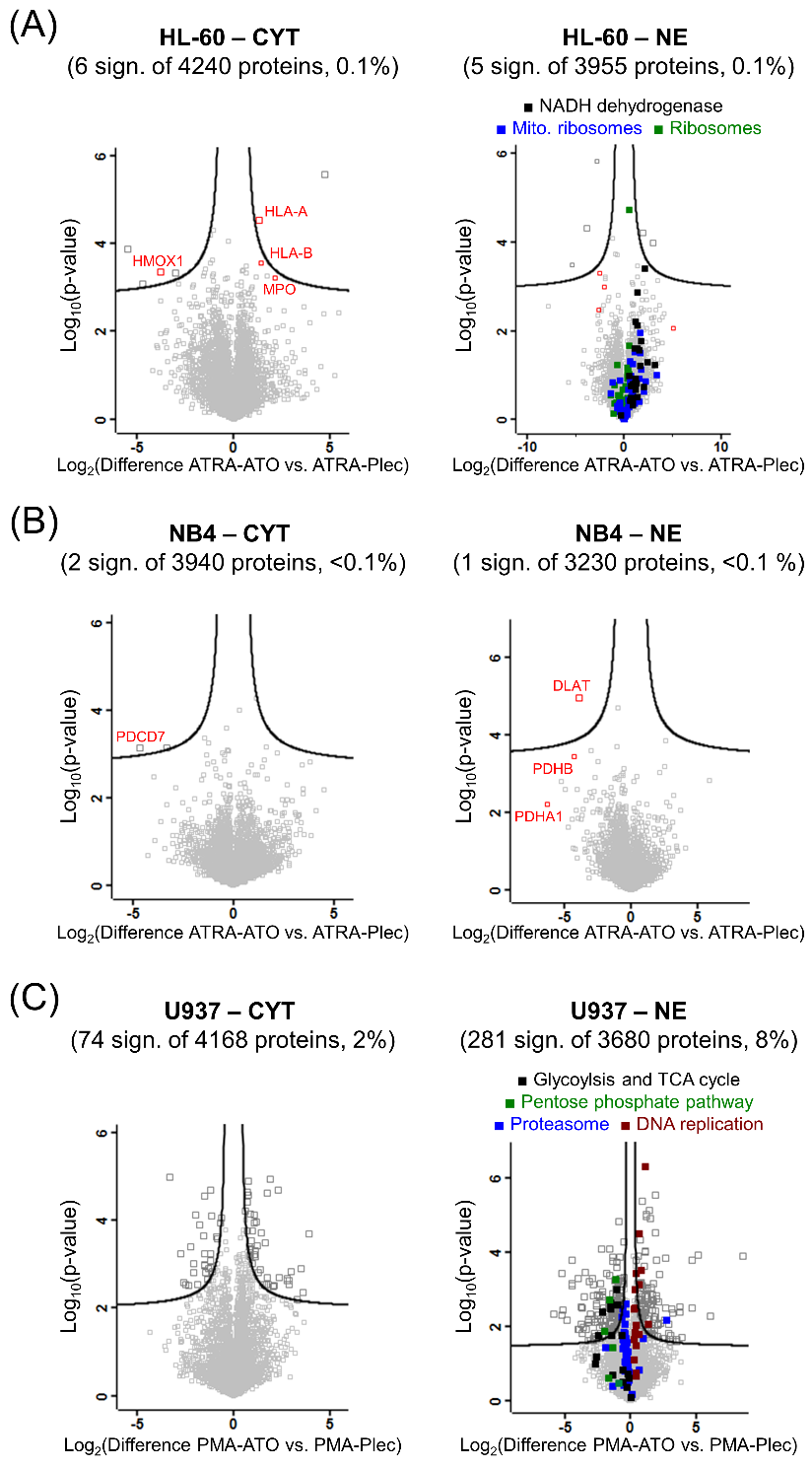


**Supplementary Figure 5.** Volcano plots comparing the co-treatments using ATRA/PMA+ATO (left side of plot) and ATRA/PMA+plecstatin-1 (right side of plot) in HL-60 **(A)**, NB4 **(B)** and U937 **(C)** cancer cell lines. Significances were calculated with Perseus (Version 1.6.6.) using an FDR = 0.05 and S0 = 0.1. Significantly regulated proteins are given dark grey, non-regulated proteins in light grey.

## Supplementary Tables

**Supplementary Table 1.** Concentrations to inhibit 50% of cell growth (IC_50_) were evaluated using the alamarBlue assay and are given in micromolar concentrations [µM]. HL-60 and NB4 cells were treated with ATRA (0.1 µM) and U937 cells were treated with PMA (0.16 µM) in combination with the metal(-loid)s.

|  |  | **IC_50_** [µM] | |
| --- | --- | --- | --- |
| **cell lines** | **FAB** | **ATO** | **plecstatin-1** |
| **HL-60** | M2 | 1^a^ | 10 ± 2 |
| **NB4** | M3 | 0.6 ± 0.3 | 7 ± 2 |
| **U937** | M5 | 0.7 ± 0.1 | 9 ± 1 |

^a^ Reference: Sumi, D., K. Suzukawa and S. Himeno (2016). Arsenic trioxide augments all-trans retinoic acid-induced differentiation of HL-60 cells. Life Sci. 149: 42-50. doi: 10.1016/j.lfs.2016.02.054.

**Supplementary Table 2.** Applied concentrations in the proteomic experiments corresponding to half-IC_50_. HL-60 and NB4 cells were co-treated with ATRA (0.1 µM) and U937 cells were co-treated with PMA (0.16 µM).

|  |  | **IC_50_** [µM] | |
| --- | --- | --- | --- |
| **cell lines** | **FAB** | **ATO** | **plecstatin-1** |
| **HL-60** | M2 | 0.5 | 5 |
| **NB4** | M3 | 0.3 | 3 |
| **U937** | M5 | 0.35 | 4.5 |
